# Concrete Naphtaline in Psoriasis

**Published:** 1842-11-26

**Authors:** 


*Concrete Naphtaline in Psoriasis.*—Dr. Emery, of the hospital St. Louis, had his attention turned to the investigation of the different products of tar as remedial agents in the treatment of skin diseases, on account of the successful results he obtained from the use of tar, and because of the unpleasant odour it gave forth. Various preparations were had recourse to, the most valuable of which proved to be the concrete naphtaline, which Dr. Emery tried in fourteen cases. In two cases, one of psoriasis gyrata, and the other lepra vulgaris, it failed in effecting any good; in the remaining twelve it proved more serviceable. Eight of these were men and four women. In two of the cases, lepra vulgaris of from fifteen months to two years' duration, arsenical and iodic preparations had been previously tried; in the younger patient the arsenic at first seemed to do good, but the improvement soon ceased. An ointment prepared with two scruples of concrete naphtaline to thirty of lard was applied, causing the scales to fall off, leaving the skin of a violet colour, with white circles around. A perfect cure was effected in six weeks, and although three months have passed since, there has not been any relapse. In four other cases the men were labouring under inveterate psoriasis; in one of them it had existed sixteen years, and had resisted arsenical, iodic and mercurial treatment. The tar ointment was had recourse to, and with decided advantage; but the man becoming impatient on account of his business, an ointment of naphtaline, twice the strength of that used in the preceding cases, was spread on compresses, and applied over the diseased parts night and morning. The man was cured in six weeks. When the ointment was applied too strong, it caused a burning heat, which was soon removed by emollient baths and poultices. The other six cases were also instances of psoriasis cured by the naphtaline ointment. Dr. Emery states that this remedy has an unpleasant odour, which soon passes off, and it is apt to irritate the skin and cause erysipelas, if it be not carefully watched.—*Ibid, from Bullétin de Thérapeutique*, July, 1842.

